# How does housing tenure mix affect residents' mental health through a social environment lens? An empirical examination from Guangzhou (China)

**DOI:** 10.3389/fpubh.2022.1024796

**Published:** 2023-01-06

**Authors:** Tianyao Zhang, Xin Li, Jiahui Liu

**Affiliations:** ^1^Department of Urban Planning and Design, School of Architecture, Harbin Institute of Technology, Shenzhen, China; ^2^College of Public Administration, Nanjing Agricultural University, Nanjing, China; ^3^Faculty of Construction and Environment, The Hong Kong Polytechnic University, Hong Kong, Hong Kong SAR, China

**Keywords:** income mix, social capital, sense of community, social control, mental health

## Abstract

This study demonstrates the mechanisms of housing tenure mix affecting residents' mental health *via* intervening community social environment within public housing practices in urban China. Using a purposive sampling data of six representative public housing estates, we used structural equation models to examine total, direct, and indirect effects of housing mix status on mental health, highlighting the intermediatory roles of social environment variables. On the whole, we find no significant impact of housing tenure mix on mental health; however, housing tenure mix thwarted mental health in a direct way but contributed to it through the mediation of social participation. Regarding the neighborhood effects, we unfold the behavioral, psychological, and socially interactional mechanisms for affecting mental health, by highlighting the direct health implications of social capital, and the mediation of sense of community and social control between social capital and mental health. Finally, we suggest to consider social effects on health grounds into mixed housing strategies in future.

## 1. Introduction

For low-income and the housing of disadvantaged populations, public housing initiative is a widely-used political intervention for ensuring and extending their right to develop healthier communities (1). While public housing is often regarded as a cause of poverty concentration and social segregation, leading to negative neighborhood effects on population health (2, 3). Theses contextual effects on public health have been documented and addressed in numerous studies (4, 5), where socialization process has been proposed as an ecological mechanism that mediate between neighborhood characteristics and individual outcomes (3, 6). To combat the undesirable social issues rooted in public housing, policymakers are keen to increase residential mix of advantaged and disadvantaged groups. Accordingly, mixed housing strategies have become widely advocated and used by politicians to achieve a social mix of population at neighborhood level in the U.S., western European countries, and Australia (2, 7). However, policymakers have not pay enough attention to how different social groups interact as neighbors and how the mixture status of a neighborhood influence individual outcomes. Although housing mix (type or tenure mix) and social mix are theoretically interrelated and have positive effects on people's lives, whether mixed housing strategies can inevitably lead to social mix and overcome negative neighborhood effects has been a subject of debate for a long time (2, 8). Particularly, there is still no clear idea as to how health issues could be addressed through mixed housing strategies within the context of western Europe (i.e., United Kingdom) (9).

The connotations associated with, and initiatives behind, mixed housing initiatives in China are distinct from their counterparts in Western countries. With the progress of housing commodification and marketization in China, mixed housing initiatives has been introduced into public housing documents since 2006, with the its initial aim being to counter housing inequalities and potential social crises (10). Driven by ambitious politically objective of providing a large number of public housing units during a short period since 2008 (i.e., 36 million, 2011–2015), housing mix policy has been operated as a tool to stimulate public housing production to control housing prices. In this sense, mixed housing strategies in China emphasize the mixture of housing tenure, implemented by developers by embedding public housing units into their commodity housing projects. Mixed housing strategies thus become an extension of economic promotion initiatives (11), and its primary objective of tackling housing and social inequities transform into an impetus for local economic growth (12). Thus, mixed housing initiatives have been accused of assuming the role of economic driver rather than social stabilizer in post-reform China (13). Within this context, the neighborhood effects of these strategies on individual health outcomes are even less noticed in political practice. Therefore, this study intended to probe the links between housing tenure mix and social mix, and their effects on social environments and the resultant influence on residents' health outcomes. Two explicit questions are addressed: (1) what is the influence of housing tenure mix on social mix and how does it structure a community's social environment? (2) How do the housing tenure mix/social mix and their resultant socialization process affect individual mental health? This study could contribute to existing literature by its exploration of the mechanisms underlying mental health implications of housing tenure mix in urban China through a social environment lens, putting forward suggestions on embedding health concerns into public housing agendas.

## 2. Theoretical background and hypotheses

### 2.1. Social effects of mixed housing strategies

Housing mix and social mix are two interrelated terms in extant research about housing and health. Mixed housing strategies are usually implemented as context-specific tools to reconfigure neighborhood characteristics, and their indirect effects related to housing tenure (i.e. wealth impacts, neighborhood effects) on health are known as increasingly important (14). There are two underlying assumptions for the social benefits of mixed housing strategies: housing mix is strongly related to social mix, and social mix could create more social opportunities (2). And they serve as the theoretical underpinning of promoting housing mix in response to social issues deriving from poverty and segregation (14). In the literature, housing mix, concerning the attributes of housing units, is consistently referred as a mixture of different housing types or tenures (2). In comparison, social mix, emphasizing more on the characteristics of people, refers to a mixture of households of different socioeconomic positions, and often measured by the mixed level of income or occupational status (2, 15). Empirically, although studies have shown that housing homogeneity that create social homogeneity could reduce social opportunities for local residents, it is still debatable whether mixed housing strategies could create a social mix community and then nurture a positive socialization process that contributes to individual health outcomes.

Most discussions about housing mix or social mix and their social effects take place within the context of public housing, where socioeconomic disadvantage is implicated as a characteristic (3, 16). Public housing estates have been criticized for their contribution to poverty concentration and its concomitants, such as limited social opportunities, lack of necessary resources and social capital, negative socialization process, stigmatization effects, social disorder and even crimes (2, 8). Thus, social mix become a key target during public housing regeneration in many Western countries (17). Literarily, studies on the housing mix, social mix and their resultant social environment generate debatable and even conflicting points of view (15, 18). Supporters highlight social benefits that mixed communities can provide, including increased social interaction and opportunities, enhanced social networks and sense of community, reduced anti-social behavior, improved reputation, and liveability of the area, etc. (17, 19), while opponents note that mixing different groups together actually reveals a loosening of social bonds, reduces community, and social cohesion, but increases conflict (20). In light of these contrasting findings, establishing whether housing mix and social mix could contribute to a higher quality of social environment within urban China requires careful examination.

### 2.2. Neighborhood social environment and mental health

The neighborhood effects of social environment on mental health has been discussed for decades in epidemiology, public health, and sociology discourses (21, 22). Extant studies on social environment and mental health suggest that social capital, strong social cohesion, high collective efficacy, and community participation could reduce the likelihood of depressive symptoms. However, lower levels of social cohesion, less cognitive social capital, and higher levels of neighborhood disorder, can all contribute to incidence of depression (23, 24). Within Chinese contexts, socialization process at local residence has been ascertained to be influential to population health, especially among particular groups. For example, social capital is found to be associated with suicidal ideation among Chinese college students (25); and local ties and trans-local ties are determinative to migrants' mental health, where social comparison and perceived social status serve as vital psychologically mediators (26). Accordingly, we focus on several frequently discussed constructs considered integral to neighborhood social environments and influential to residents' mental health, namely, social capital, social control, and a sense of community.

Arising from and implicated in everyday experiences and perceptions, social capital refers to “levels of social attachment among individuals indicative of social engagement and participation within communities” [(27), p. 104]. Many commentators consider social capital is able to affect personal health and to lead to health inequalities among different social groups (28). Scholars have various perceptions regarding the definition and measurement of social capital, and we adopt bonding social capital (social links between similar population) and bridging social capital (social links between dissimilar population) as two major dimensions, which are measured by cognitive factors (i.e., trust, social harmony) and structural factors (i.e., membership of networks), respectively (24, 29). Considering the ways in which social capital affects health, two prominent mechanisms are distinguished in literature: the compositional effect and the contextual effect (22). The former mechanism refers to that socially isolated people are more potentially to reside in neighborhoods lacking social capital, and such individuals are more easily to sustain poor health (30). For the latter, three plausible pathways are proposed: (1) by affecting people's health-related behaviors such as healthy-behavior norms, health-related information accessibility, and the exertion of social control over deviant behaviors (6); (2) by affecting people's accessibility to amenities and services which is related to the fact that cohesive neighborhoods are more likely to foster positive organizational processes that ensure good accessibility of the services and therefore are protective of health (22); and (3) by impacting psychosocial processes, as social capital could provide psychosocial resources such as self-esteem, affective support, and mutual respect that are protective of individual health (31).

As the widespread use of conventional behavioral indices to measure social capital cannot uncover the social and psychosocial processes that influence individual mental health (32), we further focus on the related psychosocial process of public housing by investigate residents' sense of community and social control. Public housing is often criticized for its inferiority in exerting sense of community and informal social control, which leads to high levels of social disorder and undermines residents' health and wellbeing (16). Sense of community can be a measure of the psychological basis upon which residents develop a willingness to intervene in community affairs (32), and it acts as the social process giving rise to informal social control (33, 34). Empirical evidence has suggested that psychological profits could accrue from experiencing a higher level of sense of community, and perceptions of health problems are linked with a lack of sense of community (35). Living in neighborhoods that lack order and social control may lead people to feel unsafe, mistrustful, powerless, isolated, angry, and anxious, all of which discourage outdoor activity and can result in mental health issues (3). By contrast, people who feel having control over their lives are more likely to increase their health conditions through health-enhancing behaviors, such as interacting with their surrounding environment in a positive way (3). Therefore, sense of community and social control are effective factors in explaining the mechanisms of social environment's influence on mental health, particularly the replenishment of the psychosocial pathways linking social capital and mental health.

### 2.3. Research hypotheses

**Figure 1:**
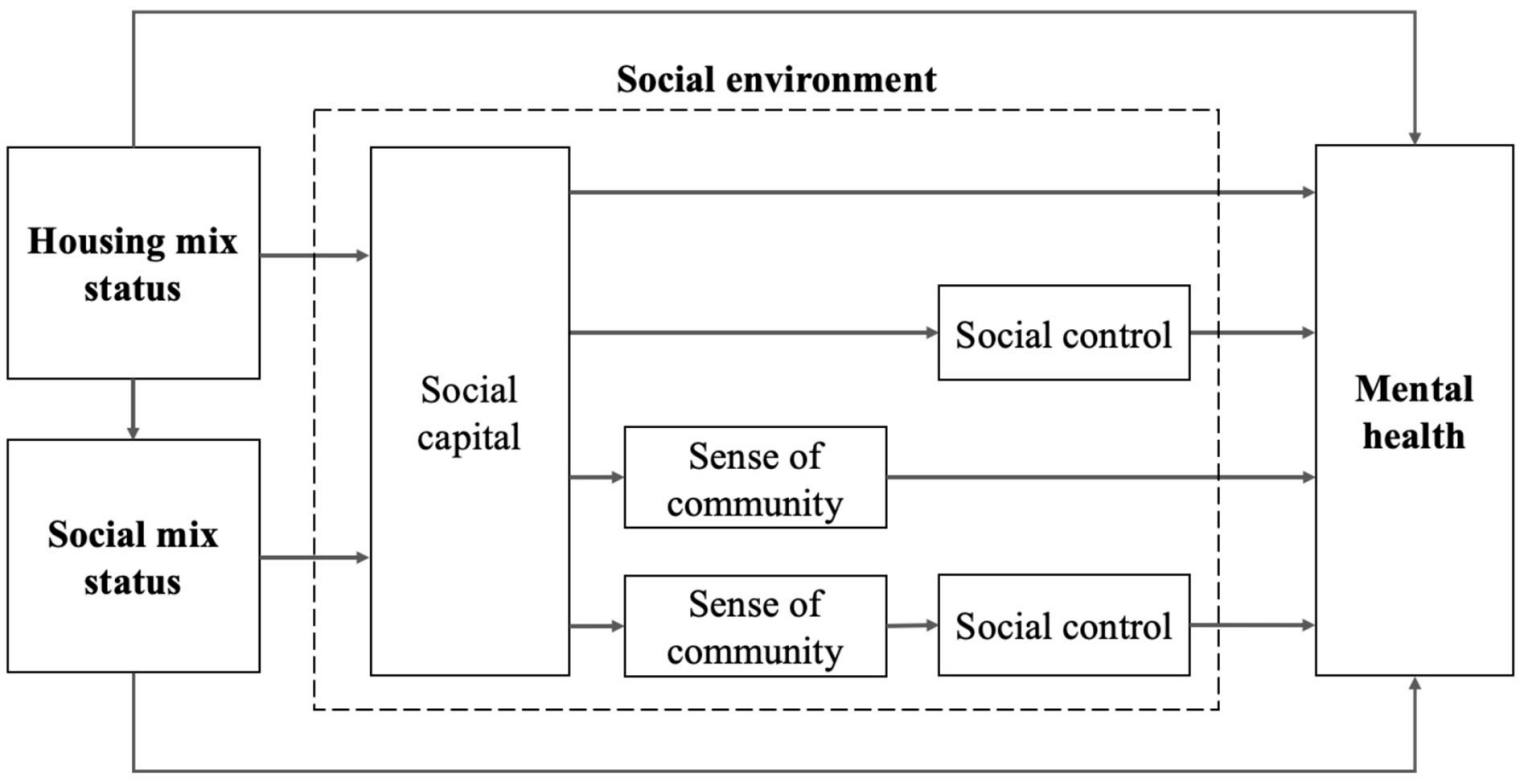
The conceptual framework.

Social environment characteristics intermediate the relationship between the levels of housing tenure mix and social mix and mental health is presented in Figure 1. Housing mix and social mix are regarded as structural determinants of health, which together shape neighborhood socioeconomic composition and cultural context. Social environment as a whole acts as an intermediate determinant between neighborhood structural factors and individual mental health. Sense of community and social control pertain to intermediate factors between the mixture status and residents' health, and social capital creates the association between housing/social mix and sense of community or social control. The following theoretical hypotheses are proposed:

H1: Housing tenure mix determines social mix and shapes the social environment characteristics.

H2: Housing tenure mix and social mix affect residents' mental health directly and indirectly.

H3: Social environment characteristics intermediate the relationship between the levels of housing tenure mix and social mix and mental health: (1) Social capital, sense of community, and social control are the mediators; (2) The mutual interactions between social capital, sense of community, and social control suggest a compound mediation mechanism.

## 3. Materials and methods

### 3.1. Case selection

**Figure 2:**
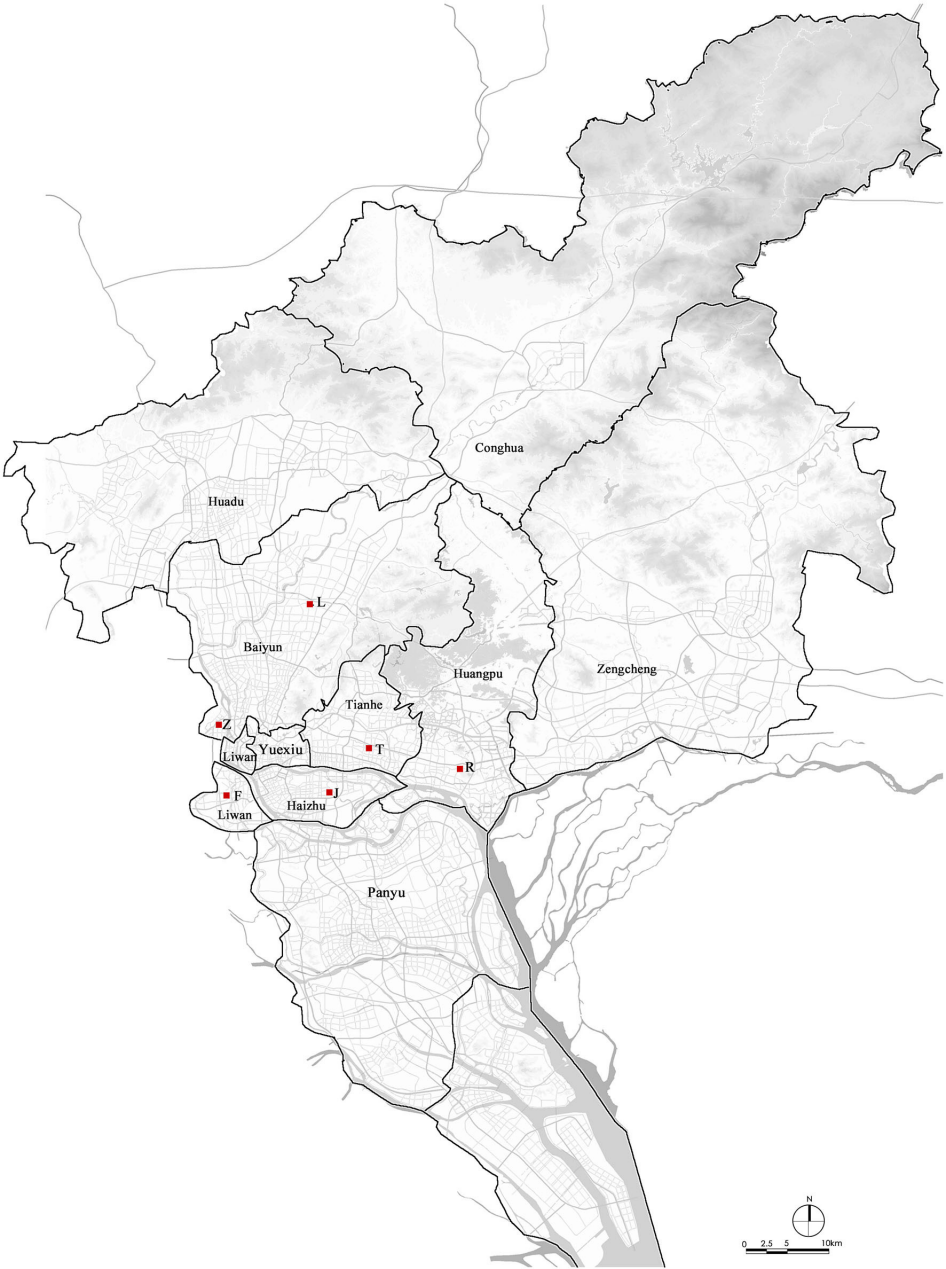
The study area.

Guangzhou was selected for the case study due to the innovative housing tenure mix the city has put in place over the last few decades. Since 2009 Guangzhou has adopted “measures for land reserve of public housing” and has regulated the lease of land by “controlling land prices but bidding on public housing.” This housing tenure mix tool has been criticized for its economic benefit driven nature, since the provision of public rental housing has become a bargaining chip between developers and the local government during the land leasing process. Considering these evolved housing tenure mix tools, we choose six typical public housing projects developed at different times in urban Guangzhou to explore the associations of housing tenure mix with residents' mental health. Selection criteria included: development period and the specific political background, location, population scale, and most importantly, the level of housing tenure mix. Finally, three tenure groups of six neighborhoods are chosen as follows (Figure 2 and Table 1):

1) Private housing dominated neighborhoods: T and J housing estates. Both of them underwent a long development periods from the 1990s to the 2010s, throughout which period dominated housing tenure type has largely transferred from subsidized owner-occupied housing (i.e., economically affordable housing) to private-owned commodity housing *via* the transaction process in second-hand house market. T community was the earliest and the largest economically affordable housing estate in Guangzhou before 2002. By now, the ratios of commodity housing units in T and J are as high as 89.86 and 80.02%, respectively.
2) Public housing dominated neighborhoods: F, L, and Z housing estates. These neighborhood are mainly comprised of public rental housing (i.e., low-rent housing) and subsidized owner-occupied housing (i.e., economically affordable housing and joint-ownership housing). Commodity housing units are excluded in F and L and only take a small percentage (11.68%) in Z. Regarding the social objectives of these projects, Z has been regarded as a pioneering public housing project since 2008, because of its efforts on tackling housing shortages and improving quality-of-life for low-income populations. F has been developed as a benchmarking sample with relative high-end decoration and application of low-carbon technologies. L is the largest public housing estates in Guangzhou by now, playing an important housing security function.
3) Tenure-mixed neighborhood: R housing estate, developed with and promoted by urban renewal process, is representative in housing both original local residents and medium and low-income populations. So this neighborhood is comprised of balanced proportions of public rental housing (32.21%), subsidized owner-occupied housing (25.52%), and private-owned relocated housing units (42.27%).

**Table 1:** Distribution of housing tenure types of the selected estates.

**Tenure groups**	**T**	**J**	**F**	**L**	**Z**	**R**
Public rental housing	10.09 (%)	13.96 (%)	32.81 (%)	61.62 (%)	60.07 (%)	32.21 (%)
Owner-occupied subsidized housing	0.04 (%)	6.02 (%)	69.19 (%)	38.38 (%)	28.25 (%)	25.52 (%)
Private housing^*^	89.86 (%)	80.02 (%)	0 (%)	0 (%)	11.68 (%)	42.27 (%)
Total units	8,888	6,528	5,935	12,298	5,918	5,992

Source: Guangzhou housing security office.

^*^Including both commodity housing and relocation housing.

### 3.2. Data collection

We administered a face-to-face questionnaire survey between 2nd and 16th June 2019. Three groups of datasets were collected: demographic and socioeconomic characteristics, perceived social environment characteristics, and mental health status. Respondents above the age of 18 who had been living in the housing estate for at least 1 year were selected, and consent was deemed to be granted by willingness to participate. Due to the difficulties in administering household surveys in gated communities, we collected data at the entrance of each housing estate by sampling one of every five passing residents. To improve sample representativeness, we referred to the ratios of each housing tenure type in each neighborhood during the sampling process, and conducted the survey during two time periods on weekdays and weekends. Survey data were collected using tablet computers. Finally, a total of 526 adults participated and 25 participants were removed from the sample due to invalid responses, such as providing exactly the same response to all items or providing incomplete responses to more than 20% of the items, leaving 501 valid surveys.

### 3.3. Variables

#### 3.3.1. Housing tenure mix and social mix

The calculation of the entropy measure—a prominent measure for the nominal variables' variation—was adopted to measure mix levels. For housing tenure mix, three housing tenure types were classified according to housing tenure and homeownership: public rental housing, subsidized owner-occupied housing, and private housing. To measure social mix status, first, four household-income groups were identified: low-income family (< 5,000 yuan), lower middle-income family (5,000–10,000 yuan), upper middle-income family (10,000–15,000 yuan), and better-off family (>15,000 yuan); second, three occupation positions were classified (job with high stability, job with low stability, unemployed). The proportions of each type of housing tenure and social status (income and occupation) relative to the total were computed, based on which the entropy measure was calculated according to Equation (1) and was standardized according to Equation (2):


(1) $$\begin{array}{c} H\left(x\right)=-\sum_{i}p_{i}\ln\;p_{i} \end{array}$$

(2) $$\begin{array}{c} H^{\prime }\left(x\right)=H\left(x\right)/\ln\;I \end{array}$$

where pi is the likelihood of an observation pertaining to category i of X (neighborhood) and pi ln pi = 0 for pi = 0, and I is the maximum number of categories that the neighborhood has—since the figure of housing/income/occupation types vary per neighborhood (2).

This entropy measure runs from 0 to 1, with 0 implying absolutely no variation and 1 standing for absolute variation. Thus, five categories were identified: (1) absolutely homogenous (0); (2) homogeneous (0.01–0.25); (3) average homogeneity (0.25–0.50); (4) average heterogeneity (0.50–0.75); (5) heterogeneous (0.75–1.00).

#### 3.3.2. Social environment: social capital, sense of community, social control

First, social capital was measured according to its two compositions: cognitive and structural social capital. Based on the social capital measurements developed by Lochner et al. (36), we developed a cohesion scale to measure the cognitive dimension of social capital and two questions on local friendship network and social participation to capture the attribute of structural social capital.

1) Cohesiveness: the cohesion scale comprised seven items on a 5-point scale. Respondents were asked to indicate how strongly they agreed or disagreed with the statements describing neighborly relations: (a) I know many neighbors in this community; (b) I frequently chat with my neighbors; (c) I visit my neighbors from time to time; (d) Neighbors can get help from each other; (e) This is a close-knit community; (f) Neighbors get along well with each other; (g) People are trustworthy in this community. The internal consistency was satisfactory (Cronbach's alpha = 0.864).
2) Local friendship network: measured by the proportion of friends within the neighborhood to total number of friends, with a higher score indicating a stronger connection to the neighborhood social network.
3) Social participation: this was measured by the average frequency with which respondents participated in three types of activities: (a) recreational activities; (b) activities organized by a residential committee or housing manager; (c) community committees. The mean score of these frequencies (rated by a 4-point scale) was calculated and higher score suggested higher frequency of social participation.

Second, a sense of community scale consisting of 14-items was constructed, and respondents were asked to answer their perceptions on a 5-point scale with statements such as “When I leave this community for a while, I miss it very much” and “I'd love to invest my resources in this community, such as money and personal efforts.” Given the internal consistency value of the items (Cronbach's alpha = 0.90), a single index of mean score was calculated, and higher score implied a greater perceived sense of community (35).

Third, social disorder, indicating that social control has broken down, was used as an indicative factor for measuring the level of collective efficacy of social control (37). Both social and physical signs can reflect the level of social disorder. To measure the level of informal social control, we asked respondents to report how often they had observed the following phenomena on a 5-point Likert-type scale: (1) physical signs (e.g., vandalism, litter, and graffiti); and (2) social signs (e.g., behavior that is harmful to society such as damaging public property and harassing neighbors; people or teenagers hanging around). Average scores were computed, with higher ones suggesting higher degree of collective efficacy of informal social control.

#### 3.3.3. Mental health

We used the variable of mental wellbeing to evaluate individuals' mental health condition. Adult Mental Health Continuum Short Form (MHC-SF, Chinese version), which shows very good internal consistency (α > 0.80) and discriminant validity in adults in various contexts, was adopted to establish residents' mental wellbeing. This scale is comprised of 14 items: three items for emotional wellbeing, six for psychological wellbeing, and five items for social wellbeing [(38); see the Supplementary Table 1]. Respondents were asked to evaluate how often they had experienced a particular feeling over the past 4 weeks according to six choices: “never,” “once or twice,” “once a week,” “two or three times a week,” “almost every day,” and “everyday.” The internal consistency of our data was satisfied (Cronbach's alpha of 0.899) and the mean score was computed to represent residents' mental wellbeing.

#### 3.3.4. Covariate variables

Socioeconomic and demographic characteristics of the respondents were collected and regarded as the control variables in this study, including age, gender, marital, education, monthly household income, and occupation status.

### 3.4. Analysis strategy

Descriptive statistics were performed to capture the overall characteristics of the samples. Then to address the first research hypothesis, quick correlation analyses were conducted between housing mix status and social mix status, and between housing mix status and social environment variables. On the one hand, we used the Mantel-Haenszel Chi-square statistic to test the associations between housing mix status and income mix status, and housing mix status and occupation mix status. On the other hand, Spearman's correlation coefficients were computed for preliminarily identification of the correlative relations between housing mix status and social environment variables.

To address the second research hypothesis, we built a structural equation model according to the conceptual framework presented in Figure 1, examining the intermediatory roles of social environment variables between housing/income mix status and mental wellbeing. Besides the direct links among housing/income mix status, social environmental variables and mental wellbeing, the mutual interactions between the components of social environment variables were also accounted for, including the interaction between cognitive social capital and structural social capital, the influence of social capital on sense of community and social control, and the influence of sense of community on social control.

Furthermore, to explore the multiple mediation of social environment variables, we used the bias-corrected deviation correction method to evaluate the total, direct, and indirect effects of two mix indicators and social environment variables on mental health. Then, the total indirect effects of housing mix status and social environment variables on mental health were broken down into specific indirect effects to identify the significant indirect paths. The analysis of mediation effect was carried out in the particular situation of repeated sampling 5,000 times by Bootstrapping, with a 95% confidence interval.

SPSS Statistics (version 24.0) and SPSS AMOS (version 24.0) software were employed to conduct correlation analysis and to construct path analysis models, respectively.

## 4. Results

### 4.1. Description of sample characteristics

The mean score for mental wellbeing was 4.50 (SD = 0.66) on a 6-point scale. Referring to the diagnostic criteria that respondents must experience “every day” (scored as 5) or “almost every day” (scored as 6) to indicate the status of flourishing mental health, the average mental health condition of the participants did not reach the flourishing level (38).

Summarizing the socioeconomic and demographic characteristics of samples, the sex ratio was 1:1, and age ranged from 18 to 86 years old, averaging 37 (SD = 12.90). The ratios of married and college-educated were 64.87 and 48.32%, respectively. Around half (49.70%) reported a monthly household income of 5,000–10,000 RMB/month, and 64.87% had formal and stable jobs.

Summarizing the characteristics of residential property, 68.46% of respondents were public rental housing tenants, 17.37% were subsidized homeowners, and the average duration of residency was 4.93 years. Different mixture levels of housing tenure were unfolded: LG (H = 0) and TD (H = 0.37) were homogeneous, while JSZ (H = 0.54), JD (H = 0.73), RD (H = 0.75), and FH (H = 0.99) were heterogeneous. To the contrast, the entropy measures of income and occupation mix suggested that all neighborhoods were socially heterogeneous.

Regarding the attributes of social capital, the average score of cohesiveness was 3.45 (SD = 0.70), suggesting a relatively cohesive social environment for cultivating social capital at the neighborhood level. The mean of closure of social network was 0.36 (SD = 0.20), and the average score for social participation was 1.93 (SD = 0.74), indicating that respondents seldom participated in collective activities. Sense of community and social control were both between a neutral and positive status (M = 3.40, SD = 0.52; M = 3.49, SD = 0.71), suggesting a slightly positive person–place emotional attachment and functional dependence, and a relatively low occurrence frequency of social disorder.

### 4.2. Preliminary correlations analyses: Housing mix, social mix, and social environment

We used Mantel-Haenszel Chi-square tests to check for the linear relation between housing tenure mix status and social mix status (the former was assigned an entropy value of housing tenure and homeownership type; the latter was assigned two entropy values of income and occupation). Results of the linear-by-linear association test of trend suggested a strong linear relationship between housing tenure mix and income mix (χ^2^ = 403.133, *p* < 0.001; Pearson's *R* = 0.898, *p* < 0.001), indicating that a higher level of housing tenure mix could contribute to a higher level of income mix. By contrast, the Mantel-Haenszel Chi-square statistic showed no significant linear relationship between housing tenure mix and occupation mix (χ^2^ = 0.905, *p* = 0.341), suggesting that housing tenure mix status had no effect on the mix level of occupation stability in this data. So we used income mix status to indicate social mix in the following statistical analyses.

Regarding the correlation between housing tenure mix and social environment, results of Spearman's correlation analysis indicated that: (1) housing tenure mix status was significantly, but weakly, associated with cohesiveness (rs = 0.182, *p* < 0.001) and social participation (rs = 0.369, *p* < 0.001); (2) but no significant correlation was found between housing tenure mix status and local friendship network (rs = −0.087, *p* = 0.053), sense of community (rs = 0.053, *p* = 0.233), and social control (rs = 0.085, *p* = 0.057). Similarly, results of Spearman's correlation analysis between income mix status and social environment variables suggested that: (1) income mix status was also significantly, but weakly, associated with cohesiveness (rs = 0.206, *p* < 0.001) and social participation (rs = 0.266, *p* < 0.001); (2) but no correlation was found between income mix status and local friendship network (rs = −0.084, *p* = 0.059) or social control (rs = 0.060, *p* = 0.182); (3) while income mix status was significantly, and very weakly, associated with sense of community (rs = 0.117, *p* = 0.009). Consistently, the correlation relationship of each variable has been visualized with the help of heatmap (Supplementary Figure 1). Accordingly, the links between housing tenure mix status and its uncorrelated social environment variables, and the links between income mix status and its uncorrelated social environment variables were not accounted for in the path analysis model.

### 4.3. Pathways between housing mix, social environment, and mental health

**Figure 3:**
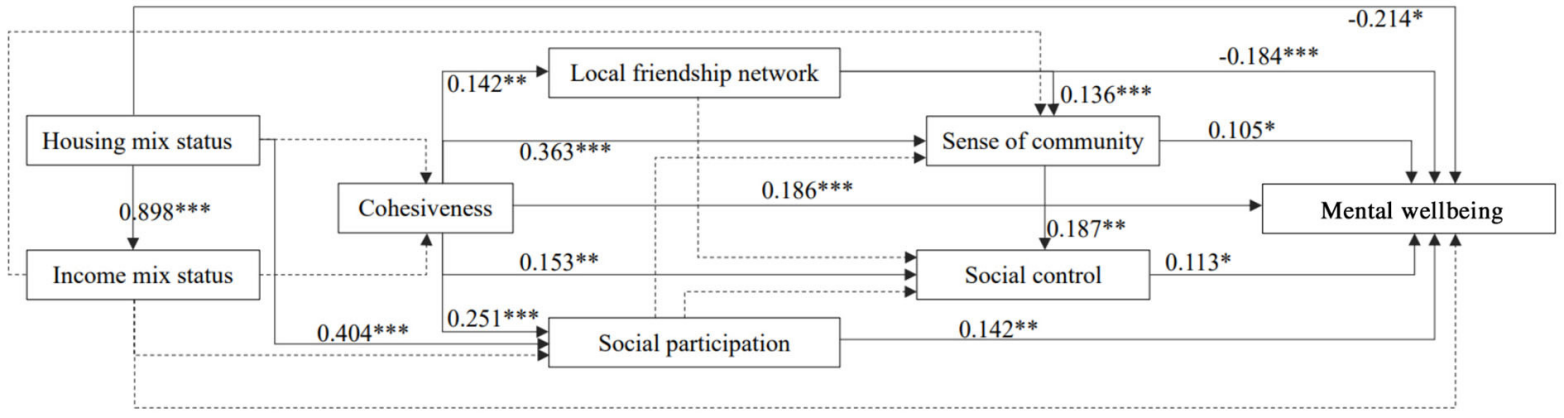
The standard coefficients of the pathway.

Goodness of fit of the path analysis model was assessed through Chi-Square/df (< 3 good, < 5 permissible; *p* > 0.05, CFI > = 0.90, GFI > 0.95, AGFI >0.90, RMSEA < 0.08), according to which our path analysis model showed satisfactory fitness for this data (χ^2^/df = 1.685, *p* = 0.034, CFI = 0.992, GFI = 0.990, AGFI = 0.957, RMSEA = 0.037). Standardized coefficients with their statistical significance are reported in Figure 3 and Table 2, showing all pairwise paths among variables in the model. The total direct and indirect effects of independent variables on mental wellbing are summarized in Table 3.

**Table 2:** Direct paths among variables of housing and social mix, social environment, and mental wellbeing (standardized estimates, N = 501).

**Path**	**Std estimate**	**S.E**.	***p***
Income mix status < –Housing tenure mix status	0.898^***^	0.005	0.000
Cohesiveness < –Income tenure mix status	0.115	0.891	0.239
Cohesiveness < –Housing tenure mix status	0.129	0.220	0.185
Social participation < –Income tenure mix status	− 0.116	0.878	0.204
Social participation < –Housing tenure mix status	0.404^***^	0.217	0.000
Sense of community < –Income tenure mix status	0.029	4.372	0.499
Social participation < – Cohesiveness	0.251^***^	0.044	0.000
Local friendship network < – Cohesiveness	0.142^**^	0.013	0.001
Sense of community < – Cohesiveness	0.363^***^	0.484	0.000
Sense of community < – Social participation	0.077	0.451	0.082
Sense of community < – Local friendship network	0.136^***^	1.586	0.000
Social control < – Cohesiveness	0.153^**^	0.049	0.002
Social control < – Local friendship network	0.012	0.153	0.791
Social control < – Social participation	0.068	0.043	0.134
Social control < – Sense of community	0.187^***^	0.004	0.000
Mental wellbeing < – Housing tenure mix status	−0.214^*^	0.202	0.024
Mental wellbeing < – Income mix status	0.132	0.802	0.160
Mental wellbeing < – Cohesiveness	0.186^***^	0.045	0.000
Mental wellbeing < – Local friendship network	−0.184^***^	0.138	0.000
Mental wellbeing < – Social participation	0.142^**^	0.041	0.002
Mental wellbeing < – Sense of community	0.105^*^	0.004	0.023
Mental wellbeing < – Social control	0.113^*^	0.041	0.010
Mental wellbeing < – Age	−0.132^*^	0.003	0.011
Mental wellbeing < – Sex	−0.009	0.055	0.829
Mental wellbeing < – Education	0.078	0.032	0.100
Mental wellbeing < – Marital status	0.016	0.060	0.734

^*^p < 0.05, ^**^p < 0.01, ^***^p < 0.001.

**Table 3:** Total, direct, and indirect effects of independent variables on mental health wellbeing (Std estimate, N = 501).

		**Housing mix status**	**Income mix status**	**Cohesiveness**	**Local friendship network**	**Social participation**	**Sense of community**	**Social control**
Mental health	Direct effect	−0.214^*^	0.132	0.186^***^	−0.184^***^	0.142^**^	0.105^*^	0.113^*^
	Indirect effect	0.231^**^	0.016	0.080^**^	0.018^*^	0.017^*^	0.021^**^	-
	Total effect	0.017	0.148	0.265^***^	−0.166^**^	0.159^**^	0.126^**^	0.113^*^

^*^p < 0.05, ^**^p < 0.01, ^***^p < 0.001.

Results suggested that housing tenure mix status impeded respondents' mental wellbeing significantly and directly (Std estimate = −0.214, *p* = 0.024), but it contributed to mental wellbeing in terms of total indirect influences (Std estimate = 0.231, *p* = 0.005). However, the total effect of housing tenure mix on mental wellbeing was not significant in this data. By contrast, income mix status had neither direct nor indirect influence on mental wellbeing (Std estimate = 0.132, *p* = 0.160; Tables 2, 3). Further, a significantly profound effect of housing tenure mix status on income mix status was confirmed (Std estimate = 0.898, *p* < 0.001), consistent with the above inference about the strong predictive power of housing tenure mix status for social mix status.

Regarding the total, direct, and indirect effects of social environment variables on mental wellbeing: (1) on the whole, cohesiveness played the most important protective role of enhancing mental wellbeing (Std estimate = 0.265, p < 0.001), followed by social participation (Std estimate = 0.159, *p* = 0.003), sense of community (Std estimate = 0.126, *p* = 0.009), and social control (Std estimate = 0.113, *p* = 0.012); whereas local friendship network had an adverse effect on mental wellbeing (Std estimate = −0.166, *p* = 0.001); (2) cohesiveness, social participation, and sense of community produced positive direct and indirect effects on mental wellbeing; local friendship network impeded mental wellbeing directly (Std estimate = −0.184, *p* < 0.001), but slightly contributed to mental wellbeing by indirect pathways (Std estimate = 0.080, *p* = 0.049), and social control contributed to mental wellbeing directly (Table 3).

Furthermore, we essentially broke down the total indirect effects of housing tenure mix on mental wellbeing into the specific indirect effects that were significant and non-significant. According to the standardized estimate of the path coefficients, the most important indirect path linking housing mix status to mental wellbeing flows through social participation (Std estimate = 0.121, *p* = 0.004). The full path linking housing tenure mix status, social participation, sense of community, social control, and mental wellbeing was also significant, but the coefficient was quite small (Std estimate = 0.001, *p* = 0.049), indicating very weak indirect impact (Supplementary Table 2).

Regarding specific indirect effects of social environment variables on mental wellbeing, 18 paths were examined. Although effects were slight, we found that: (1) structural social capital, sense of community, and social control played significant mediating roles between cohesiveness and mental wellbeing; (2) sense of community played protective mediating roles between social capital and mental wellbeing; (3) social control played protective mediating roles between cohesiveness and mental wellbeing, and between sense of community and mental wellbeing (Supplementary Table 3). We thus confirm the compound paths between social environmental variables and mental wellbeing, which are induced by the mutual interactions among social capital, sense of community, and social control.

## 5. Discussion

### 5.1. Social effects of housing tenure mix

Regarding Hypothesis 1, we proved that housing tenure mix had profound impacts on social mix partially, and moderate effects on social capital partially as well. Given the strong positive association between housing tenure mix and income mix, we believe that housing tenure mix can promote social mix partially. This intrinsically causal association could be driven by the original intention of Chinese housing mix policy, which primarily focused on adjusting housing prices through combining public housing and commodity housing units (10, 39). Diversified tenure types are encouraged by the government's involvement in the owner-occupied section, such as subsidized housing owners, housing owners in *Danwe*i communities, and tenants of public rental housing, making it reasonable to construct a mixture of families with different income statuses (40). However, diverging from some findings in the Western context in terms of the positive effects of housing tenure mix on occupation mix (15), we found no association between them. This may indicate that housing tenure mix policy has played little role in increasing social opportunities or promoting social mobility, since individuals' employment position is often considered to adequately indicate their social opportunities (2).

This study also adds additional evidence on the positive effects of housing tenure mix on structural social capital, by noting that housing tenure mix may lead to more opportunities for social interactions and participations. Disadvantaged and declining neighborhoods usually hinder residents' willingness to take part in collective activities, on the countrary, better-off community socioeconomic status is a strong determinant of social participation (41). Housing tenure mix has significantly improved the socioeconomic status of the communities in our study, since nearly 50% of our respondents were college-educated and nearly half reported a household income of 5,000–10,000 RMB per month. And the improved socioeconomic position could contribute to increased structural social capital (20). Responding to the previous accusation that housing tenure mix serves as an economic driver rather than a social stabilizer in public housing development (13), we may refute this argument partially by demonstrating the positive influence of housing tenure mix on social participation, which is an important component comprising social capital. But it should also be noted that the improved community socioeconomic position could probably be caused by the inflow of target groups such as the newly emerging middle class, rather than as a natural result of local development (11). Therefore, the selection effect of housing tenure mix on forming community socioeconomic structures should be considered when determining the effectiveness of housing mixed strategy and requires further research.

### 5.2. Mental health effects of housing tenure mix

In relation to Hypothesis 2 and 3, that on the whole, neither housing tenure mix nor income mix affected mental health significantly; rather, housing tenure mix thwarted mental health in a direct way but contributed to it in an indirect way through promoting social participation. Housing tenure mix impaired mental health directly, which differs from some Western evidence supporting the hypothesis that mixed tenure can benefit individual of various dimensions (20). Considering the trend of housing capitalization in urban China, we suspect that housing tenure mix makes it difficult for mixed community residents to develop positive psychological cognition due to the poverty metaphor attached to public housing. Unlike the West's practice whereby public housing units are increasingly being designed to be externally indistinguishable from market-rate units (42), the public housing units in our study were explicitly identified as such by signs on the building facades, so such tenants could easily be differentiated from commodity housing residents. Further, those living in public housing are often stigmatized by the media and in the minds of the public in general for their reliance on government subsidies and perceived self-destructive and non-mainstream behaviors (33, 42). On this basis, various social conflicts exist between public housing tenants and commodity housing homeowners regarding public facilities and open space, property fees, and related management services, which has been reported often in local news. Thus, the poverty and backwardness metaphor of public housing and its related social stigma undermine the development of positive psychological cognition toward mixed-housing communities. Further, this study confirms the mediating role of social capital between housing tenure mix and mental health, of which underlying social mechanisms are demonstrated as follows.

### 5.3. Social mechanisms for mental health

Considering the intermediatory role of social environment variables, this study identifies the direct health impact of social capital, as well as mediation of sense of community and social control between social capital and mental health. Accordingly, the behavioral, psychological, and mutually interactional mechanisms of social environment variables influencing mental health were revealed.

Social participation, as a health-related behavior, improves mental health on a behavioral mechanism basis. Consistent with established arguments, social participation is helpful for forming social networks that communicate information and share resources, providing social support, and establishing social norms, which are conducive on health grounds (9, 31). Increasing social participation means more opportunities to interact with diverse neighbors, and one can obtain more information, have more opportunities to gather resources and affective support, and gain mutual respect and self-esteem during these social activities. In comparison, cohesiveness and sense of community were protective for mental health on a psychological mechanism basis, such as through reducing a person's risk of social exclusion (43), increasing emotional sustenance to alleviate the emotional impacts of stressors (44), and providing individuals with meaningful social connections and enhancing self-esteem (22).

Behavioral and psychological mechanisms are usually intertwined with each other, affecting residents' health synergistically. Specifically, cohesiveness could protect individual health by promoting emotion sustaining behaviors and instrumental aid from neighbors (44). Further, a lack of social control could result in cognition of a threatening environment and resultant feelings of unsafe, powerless, isolated, anxious, and depressed, as well as discourage health-enhancing behaviors, which all impede residents' mental health (3). During the field trips, a significant amount of litter, abandoned furniture, vandalism, and run-down buildings were observed in JD and TD, which may suggest that the residents may not respect the properties they live in and these communities showed incapability of dealing with local problems (45). Thus, lacking social control can threat residents' mental health in two ways: first, signs of disorder could induce the psychophysiological response it engenders. Repeated exposure to disorder and a threatening environment put people under frequent and intense stress response status, which can, in turn, erode mental health. Second, people whose lives are under control have a high possibility to take health control and health-enhancing behaviors, such as enhancing physical activities and interacting with their surrounding environment in a positive way (3, 45).

Based on the transactions between social environment variables, socially interactional mechanisms are proposed, highlighting the mediation of sense of community and social control between social capital and mental health. Social capital is found to be greatly conducive to the growth of sense of community, which echoes the established viewpoint that sense of community, as integral to sustaining a community, is a correlate of social capital (46). Sense of community has been ascertained to be affected by several aspects of social environment, including neighborhood cohesion and satisfaction, community ties and support, and participation in community organizations (34). Our findings support this viewpoint by revealing that cohesiveness and connections with local social networks can contribute to sense of community.

Social control intermediates the relationship between cognitive social capital and mental health. Cohesiveness could assist residents maintain informal social control by providing mutual support among neighbors and reducing the number and extent of the stressors that residents perceive in neighborhoods (47). Cohesiveness is also a precursor to community problem-solving because it can increase the likelihood that residents care about the community and are able to achieve consensus in relation to both acknowledging and addressing community problems (48). However, structural social capital had little impact on social control in this study. Although previous studies propose that local friendship networks could promote social control by helping residents to recognize strangers and enabling guardianship behaviors (49), and social participations can mitigate victimization and delinquency (50), this study finds that having local friends and participating in collective activities may not be enough to enable the communities to exercise social control. A possible explanation may be that: their seeming irrelevance to social control may be derived from the spirit of the golden mean as expressed by the Chinese philosophy of Confucianism (namely Middle, “中庸” in Chinese). Following the standard of moderation, people would act prudently when interfering in the behavior of others, which explains the non-existent impact of structural social capital on social control in this study.

Also, social control serves as a mediator between sense of community and mental health. Sense of community can benefit the degree to which residents work together on common public problems, fostering the community's ability to exercise informal social control (51). For the declining appearance of the residential environment, sense of community may nurture the community's ability of improving the living environment and benefiting residents' mental health accordingly.

Lastly, we found contradictory effects of connections with local friendship networks on mental health between direct and indirect pathways. Although connections with local friends indirectly contributed to mental health through increasing sense of community, there was much stronger negative associations between local friendship networks and mental health. Concerning the measurement of local social networks we used, which indicates the diverse social, cultural, and ecological contexts where residents are embedded, higher level of local friendship network refers to lower level of friendship heterogeneity (52). People who depend on a local friendship network may have less support than those with diverse and heterogenous social networks, which is not conducive to protecting personal health (53). It is thus reasonable that the social network developed within the mixed communities may be inadequate and not strong enough to fully support the residents.

## 6. Conclusion

With empirical evidences from Guangzhou, we ascertained that housing tenure mix influenced residents' mental health in either direct or indirect ways with opposite effects: it threatened residents' mental health directly; meanwhile, it improved neighborhood social capital *via* increasing the opportunities of social participation, which further contributed to residents' mental health. For the underlying social mechanisms affecting mental health, we unfolded that: on the one hand, the positive psychosocial process engendered by cohesiveness and sense of community can protect individuals from psychological issues. Cohesiveness and sense of community determine better social control, thereby giving rise to improved mental health, albeit indirectly. On the other hand, social participation and social control play proactive roles in mental health on a behavioral mechanism basis. Considering social capital as a complete indicator, the contradictory health effects of structural social capital are displayed, suggesting the potentially complicated influence of social capital on health.

To summarize, future housing mix strategies should look on their implications for the positive social environment that would be proactive in fostering residents' mental health. We propose that there will be significant mental health benefits if housing mix intervention paid more attention on the improvement of the quality of social environment, specifically, promoting neighborhood cohesion, strengthening a sense of community, and forming high level of informal social control. Additionally, we propose that there should be an “ideal” level of housing tenure mix for maximizing social and health benefits, a question which should be further explored and determined for the future mixed housing strategies.

## Data availability statement

The raw data supporting the conclusions of this article will be made available by the authors, without undue reservation.

## Author contributions

TZ and XL: conception and design of the study and drafting the manuscript. TZ: acquisition of data. TZ and JL: analysis and/or interpretation of data. TZ, XL, and JL: revising the manuscript critically for important intellectual content. All authors contributed to the article and approved the submitted version.
