# Supplementary material for: How does housing tenure mix affect residents' mental health through a social environment lens? An empirical examination from Guangzhou (China)
Source: Front Public Health. 2023 Jan 6;10:1024796. doi: 10.3389/fpubh.2022.1024796 (PMC9859634; doi:10.3389/fpubh.2022.1024796)
Supplement: Supplementary file 1 [file Data_Sheet_1.pdf]

Supplementary Material

1 Supplementary Figures and Tables

1.1 Supplementary Tables

Table 1 Adult Mental Health Continuum Short Form (MHC-SF) (ages 18 or older)

During the past month, how often did you feel …	Never	Once or twice	About once a week	About 2 or 3 times a week	Almost everyday	Everyday
1. happy						
2. interested in life						
3. satisfied with life						
4. that you had something important to contribute to society						
5. that you belonged to a community (like a social group, or your neighborhood)						
6. that our society is a good place, or is becoming a better place, for all people						
7. that people are basically good						
8. that the way our society works makes sense to you						
9. that you liked most parts of your personality						
10. good at managing the responsibilities of your daily life						
11. that you had warm and trusting relationships with others						
12. that you had experiences that challenged you to grow and become a better person						
13. confident to think or express your own ideas and opinions						
14. that your life has a sense of direction or meaning to it						

Source: Keyes, C. L. M. (2009). Atlanta: Brief description of the mental health continuum short form (MHC-SF). Available: http://www.sociology.emory.edu/ckeyes/. [On–line, retrieved 02/03/2019].

Table 2. Specific indirect effects of housing mix status on mental health wellbeing

Specifical indirect path	Std Estimate	Bootstrapping 95% Confidence Interval		p
		Lower limit	Upper limit	
Housing mix status ---> Social participation ---> Mental health	.121**	.034	.251	.004
Housing mix status ---> Social participation ---> Sense of community ---> Mental health	.007	.000	.026	.058
Housing mix status ---> Social participation ---> Sense of community ---> Social control ---> Mental health	.001*	.000	.006	.049
Housing mix status ---> Social participation ---> Social control ---> Mental health	.007	-.001	.025	.084

Note: *p<0.05, **p<0.01, ***p<0.001.

Table 3. Specific indirect effects of social environment variables on mental health wellbeing

Specific indirect path	Std Estimate	Bootstrapping 95% Confidence Interval		p
		Lower limit	Upper limit	
Cohesiveness---> Local friendship network ---> Mental wellbeing	-0.025**	-0.049	-0.009	.001
Cohesiveness---> Local friendship network ---> sense of community ---> Mental wellbeing	0.002*	0.000	0.006	0.011
Cohesiveness---> Local friendship network ---> Sense of community ---> Social control ---> Mental wellbeing	0.000	0.000	0.002	0.005
Cohesiveness---> Local friendship network ---> social control ---> Mental wellbeing	0.000	-0.001	0.003	0.659
Cohesiveness ---> Social participation ---> Mental wellbeing	0.033**	0.011	0.063	0.003
Cohesiveness ---> Social participation ---> sense of community ---> Mental wellbeing	0.002	0.000	0.007	0.069
Cohesiveness ---> social participation ---> sense of community ---> social control ---> Mental wellbeing	0.000	0.000	0.002	0.056
Cohesiveness ---> social participation ---> social control ---> Mental wellbeing	0.002	0.000	0.007	0.089
Cohesiveness ---> sense of community ---> Mental wellbeing	0.036*	0.005	0.075	0.022
Cohesiveness ---> sense of community ---> social control ---> Mental wellbeing	0.007**	0.002	0.017	0.005
Cohesiveness ---> social control ---> Mental wellbeing	0.016*	0.003	0.041	0.011
Social participation ---> sense of community ---> Mental wellbeing	0.007	-0.001	0.025	0.075
Social participation ---> sense of community---> social control---> Mental wellbeing	0.001	0.000	0.005	0.056
Social participation ---> social control---> Mental wellbeing	0.007	-0.001	0.022	0.097
Local friendship network ---> sense of community ---> Mental wellbeing	0.046*	0.007	0.113	0.016
Local friendship network ---> sense of community ---> social control ---> Mental wellbeing	0.009**	0.002	0.025	0.006
Local friendship network ---> social control ---> Mental wellbeing	0.004	-0.028	0.048	0.699
Sense of community ---> social control ---> Mental wellbeing	0.002**	0.000	0.004	0.007

Note: *p<0.05, **p<0.01, ***p<0.001.

1.2 Supplementary Figures

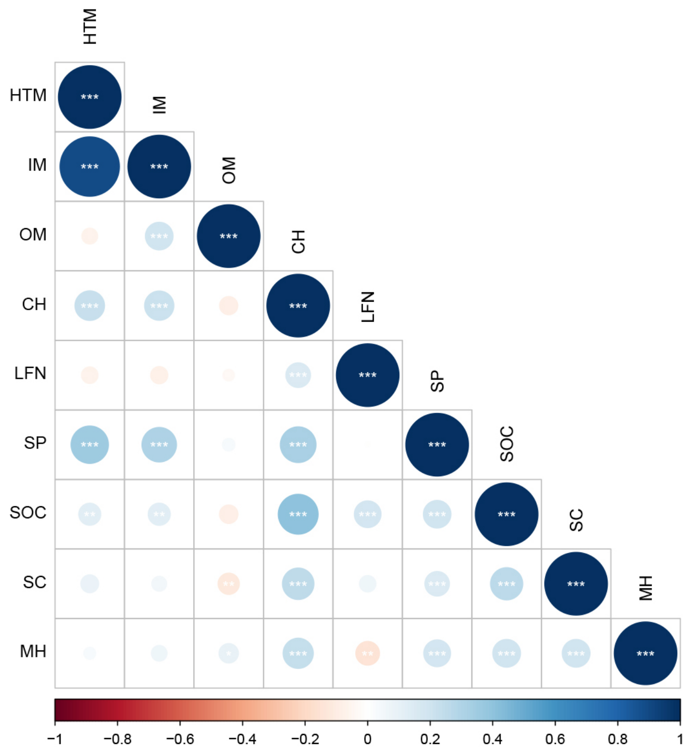

Supplementary Figure 1. Heatmap of correlation relationship of each variable

HTM: housing tenure mix; IM: income mix; OM: occupation mix CH: cohesiveness; LFN: local friendship network; SP: social participation; SOC: sense of community; SC: social control; MH: mental health.

The size of the bubbles and shade of color indicate the strength of the correlation.
